# Both MAPK and STAT3 signal transduction pathways are necessary for IL-6-dependent hepatic stellate cells activation

**DOI:** 10.1371/journal.pone.0176173

**Published:** 2017-05-04

**Authors:** Polina Kagan, Maya Sultan, Irina Tachlytski, Michal Safran, Ziv Ben-Ari

**Affiliations:** 1 Liver Research Laboratory, Sheba Medical Center, Ramat Gan, Israel; 2 The Sackler School of Medicine, Tel Aviv University, Tel Aviv, Israel; 3 Liver Disease Center, Sheba Medical Center, Ramat Gan, Israel; University of Navarra School of Medicine and Center for Applied Medical Research (CIMA), SPAIN

## Abstract

**Background:**

During liver injury, hepatic stellate cells (HSCs) can undergo activation and transform into alpha-smooth muscle actin (αSMA)-expressing contractile myofibroblast-like cells, leading to deposition of excessive scar matrix. We have recently demonstrated that depletion of adenosine deaminase acting on double-stranded RNA (ADAR1) from mouse hepatocytes leads to HSC activation and induction of inflammation and hepatic fibrosis that is mediated by interleukin 6 (IL-6). Our aim was to identify and characterize the molecular pathways involved in the direct, inflammation-independent activation of HSCs by IL-6.

**Methods:**

Primary HSCs were isolated from mouse livers. mRNA levels of αSMA and Col1a were analyzed using qRT-PCR. Protein levels of αSMA, MAPK, p-MAPK, p38, p-p38, STAT3 and p-STAT3 were assessed by Western Blot analysis. The effect of specific signal transduction pathway inhibitors (i.e., SB203580 (P-38 inhibitor), U0126 (MAPK inhibitor), S3I-201 (STAT3 inhibitor) and Ruxolitinib (Jak1/2 inhibitor)) was also studied.

**Results:**

Primary HSCs treated with IL-6 demonstrated upregulation of αSMA and Col1a mRNA levels as well as increased αSMA protein levels. Moreover, the phenotypic transition of quiescent HSCs toward myofibroblast-like cells was noted upon administration of IL-6 and not in untreated samples. In addition, the phosphorylation levels of p38, MAPK and STAT3 increased 30 minutes after treatment, and was followed by a decline in the phosphorylation levels 2–4 hours post-treatment. However, addition of specific signal transduction pathway inhibitors curbed this effect, and resulted in αSMA and Col1a expression levels similar to those measured in untreated control samples.

**Conclusion:**

IL-6 can directly induce the transition of HSCs toward myofibroblast-like cells. The effect is mediated by the activation of both MAPK and JAK/STAT signaling pathways. Elimination of either MAPK or JAK/STAT signaling pathways inhibits HSC stimulation. These results might pave the road toward the development of potential therapeutic interventions for hepatic fibrosis.

## Introduction

Liver fibrosis is a reversible wound healing response to either acute or chronic cellular injury, and reflects the balance between liver repair and scar formation. Following liver injury, hepatic stellate cells (HSCs) can undergo activation and transform into myofibroblast-like cells. This activation is characterized by vitamin A reservoir release, high proliferation rate, synthesis of a type I collagen-rich fibrotic matrix, expression of the cytoskeletal protein α smooth muscle actin (αSMA), the most abundant extracellular matrix protein [1]. Progressive deposition of matrix leads to structural and functional disturbance of hepatic function [2]. During this activation process, HSCs also release pro-inflammatory, pro-fibrogenic and pro-mitogenic stimuli that act in an autocrine and paracrine manner [3].

Stellate cell activation is a tightly programmed response occurring in a reproducible sequence. The early stage, known as initiation, is associated with transcriptional events and induction of immediate early genes, as well as rapid phenotypic changes. These early changes are likely to result from the paracrine effect of all neighboring cell types, including sinusoidal endothelial cells, Kupffer cells, hepatocytes, platelets, and leukocytes [4]. PDGF is the most potent activator of HSCs, while other proteins, such as VEGF, thrombin and its receptors, EGF, TGFα and bFGF, have been shown to also play a role in HSC activation and proliferation [5,6].

Interleukin-6 (IL-6) is a potent pleiotropic cytokine that exerts multiple functions in the body. Under physiological conditions, it is essential for proper hepatic tissue homeostasis, liver regeneration, infection defense and fine-tuning of metabolic functions [7]. However, its role in liver fibrosis induction remains an issue of controversy. Choi et al. showed that 13 weeks of IL-6 injections, twice a week, induced hepatic inflammation and collagen synthesis in rats [8]. IL-6 expressed from Kupffer cells up-regulated the expression of Col1a and directly activated αSMA expression in HSCs [9,10]. In line with these reports, IL-6-deficient mice treated with Carbon tetrachloride CCl4, a known inducer of liver fibrosis, for 12 weeks, presented fewer fibrotic changes [11]. However, other studies have demonstrated that IL-6 knockout (KO) mice were more susceptible to fibrosis development in a CCl4 hepatic injury model, suggesting a central role for IL-6 in reducing CCl4-induced acute and chronic liver injury and fibrosis [12]. Streetz et al. also suggested a protective role of IL-6/gp130, the signal transducer common to IL-6 family cytokine-dependent pathways, in nonparenchymal liver cells during fibrosis progression in chronic liver diseases [13].

IL-6 is mediating its signal transduction through the activation of the STAT1/STAT3 and/or the MAPK pathways [13,14]. In our previous work regarding liver inflammation and fibrosis in hepatocyte-specific ADAR1-depleted mice, we demonstrated that IL-6 is the mediator of HSC activation in this model ^15^ In the current study, we further investigated the significance of IL-6 as a direct, inflammation-independent stimulator of HSC differentiation toward myofibroblast-like cells and characterized the molecular pathways that are activated in this process.

## Materials and methods

### Primary hepatic stellate cell isolation

All experiments were carried out in accordance with the institutional guidelines for animal care. The experimental protocol was approved by the Chaim Sheba Medical Center ethics committee. ICR white mice (Harlan) were sacrificed using isoflurane, USP Terrell ^TM^ (Piramal). Livers were dissected and washed using Gey's balanced salt solution (GBSS) as buffer. The livers were incubated in a 37°C bath with GBSS200 (GBSS, supplemented with collagenase (Enco), pronase (Dyn Diagnostics) and CaCl_2_), for 15 minutes. GBSS100 (GBSS, supplemented with collagenase (Enco), pronase (Dyn Diagnostics), CaCl_2_ and DNase I (Sigma)) was then added to the suspended cells for 30 minutes. Afterwards, the liver extract was filtered through a steel net and divided into 50 ml tubes and centrifuged at room temperature (2000 rpm, 7 min). The supernatant was discarded and the pellet was suspended in 7 ml OptiPrep ^TM^ (Sigma) diluted in GBSS (1:6). After the pellet was suspended additional volume of GBSS buffer (5 ml) was slowly added. The tubes were centrifuged (room temperature, 14,000 rpm, 20 min, without break). The HSC layer was collected to a new tube and 20 ml DMEM were added. The cells were then centrifuged (room temperature, 2000 rpm, 7 min), the supernatant was discarded and the pellet was washed again with 20 ml DMEM. The pellet was re-suspended in 10 ml 10% FBS DMEM, to a cell density of 4*10^6^ cells/ml. Cells were cultured in DMEM, supplemented with 10% FBS, 1% penicillin: streptomycin and 1% glutamine, at 37°C, in a humidified incubator with 5% CO2.

### IL-6 signal pathway inhibition

Freshly isolated primary HSCs were treated with increasing concentrations of MAPK or STAT3 signal transduction pathway inhibitors U0126 (Cell Signaling), SB203580 ((SelleckChemical), S31-201 (Santa Cruz) and Ruxolitinib (InvivoGen) in DMEM containing 2% FBS. After 1 hour 100ng/ml IL-6 were added to the medium. Cell were harvested after additional 24 hours.

### RNA extraction and reverse transcription

Total RNA was extracted from cells using the TRI Reagent® (Sigma), according to the manufacturer's protocol. RNA concentration was determined with the NanoDrop spectrophotometer (Thermo Scientific). cDNA was prepared by reverse transcription (High Capacity cDNA Reverse Transcription Kit, Applied Biosystems) of 2μg total RNA, according to the manufacturer's instructions.

### qRT-PCR

Quantitative real-time polymerase chain reaction was performed using Fast SYBR® Green PCR Master Mix (Applied Biosystems), with the specific primers and probes listed in Table 1. The reaction was performed with the Applied Biosystems StepOne™ Real-Time PCR System. The relative quantification of mRNA analyses were performed using the ΔΔCT method (comparative ΔCT), with HPRT or 18S as the internal control gene for normalization. Each reaction was performed in triplicates and each experiment was performed at least three times.

**Table 1:** Sequences of primers used in this study (F-forward; R- reverse).

	Forward	Reverse
m Col1a	ACTGGAAGAGCGGAGAGTAC	GCACAGACGGCTGAGTAG
m αSMA	CTGCCGAGCGTGAGATTG	AGGCAGTTCGTAGCTCTTCT
m HPRT	TACTGTAATGATCAGTCAACG	GGTCCTTTTCACCAGCA
m 18S	ACCCGTTGAACCCCATT	TCCAATCGGTAGTAGCG

### Protein extraction

Proteins were extracted from cells using RIPA buffer (Sigma), supplemented with protease inhibitor (Roche) and phosphatase inhibitor cocktail 2 and 3 (Sigma), followed by quantification using the BCA kit (Pierce), according to the manufacturer’s protocol.

### Western blotting

Proteins were separated on a 10% SDS gel, and then transferred to nitrocellulose membranes (Whatman). The membranes were probed with anti-αSMA monoclonal antibody (1:1000) (Sigma Aldrich), anti-desmin polyclonal antibodies (1:1000) (Cell Signaling), anti-p38 MAPK polyclonal antibodies (1:1000) (Cell Signaling), anti-phospho-p38 MAPK (Thr180/Tyr182) monoclonal antibody (1:2000) (Cell Signaling), anti-p44/42 MAPK (Erk1/2) monoclonal antibody (1:1000) (Cell Signaling), anti-phospho-p44/42 MAPK (Erk1/2) (Thr202/Tyr204) antibody (1:2000) (Cell Signaling), anti-STAT3 antibody (1:1000) (Cell Signaling) or anti-p-STAT3 antibody (1:2000) (Cell Signaling), followed by incubation with an HRP-conjugated secondary antibody (1:10,000) (Jackson ImmunoResearch,) and later with ECL chemiluminescent substrate (Pierce Waltham). Relative quantification of the proteins was performed using the Image J software.

### Statistical analysis

Comparative means were calculated using the Student's t-test (Analysis of Variance), with significance level set at *p* ≤ 0.05.

## Results

### IL6 stimulate HSCs differentiation toward myofibroblast like cells

**Fig 1:**
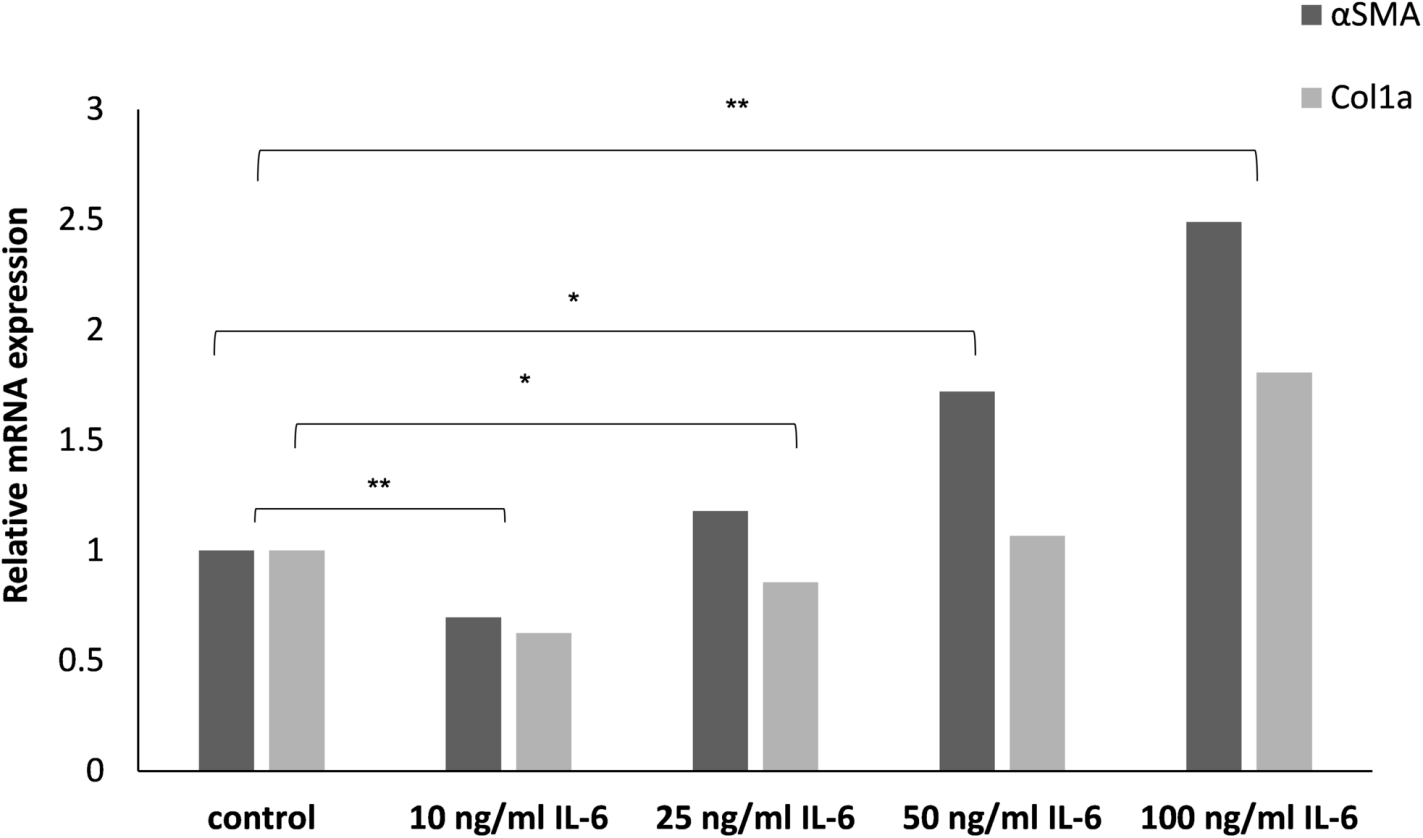
IL-6 directly stimulates HSC activation. Freshly isolated primary HSCs were treated with increasing concentrations of IL-6 in 2% FBS DMEM. HSCs were harvested 24 h later and the levels of αSMA and Col1a were evaluated using qRT-PCR. qPCR relative mRNA expression levels are shown. Error bars indicate standard deviation, * *p* < 0.05.

**Fig 2:**
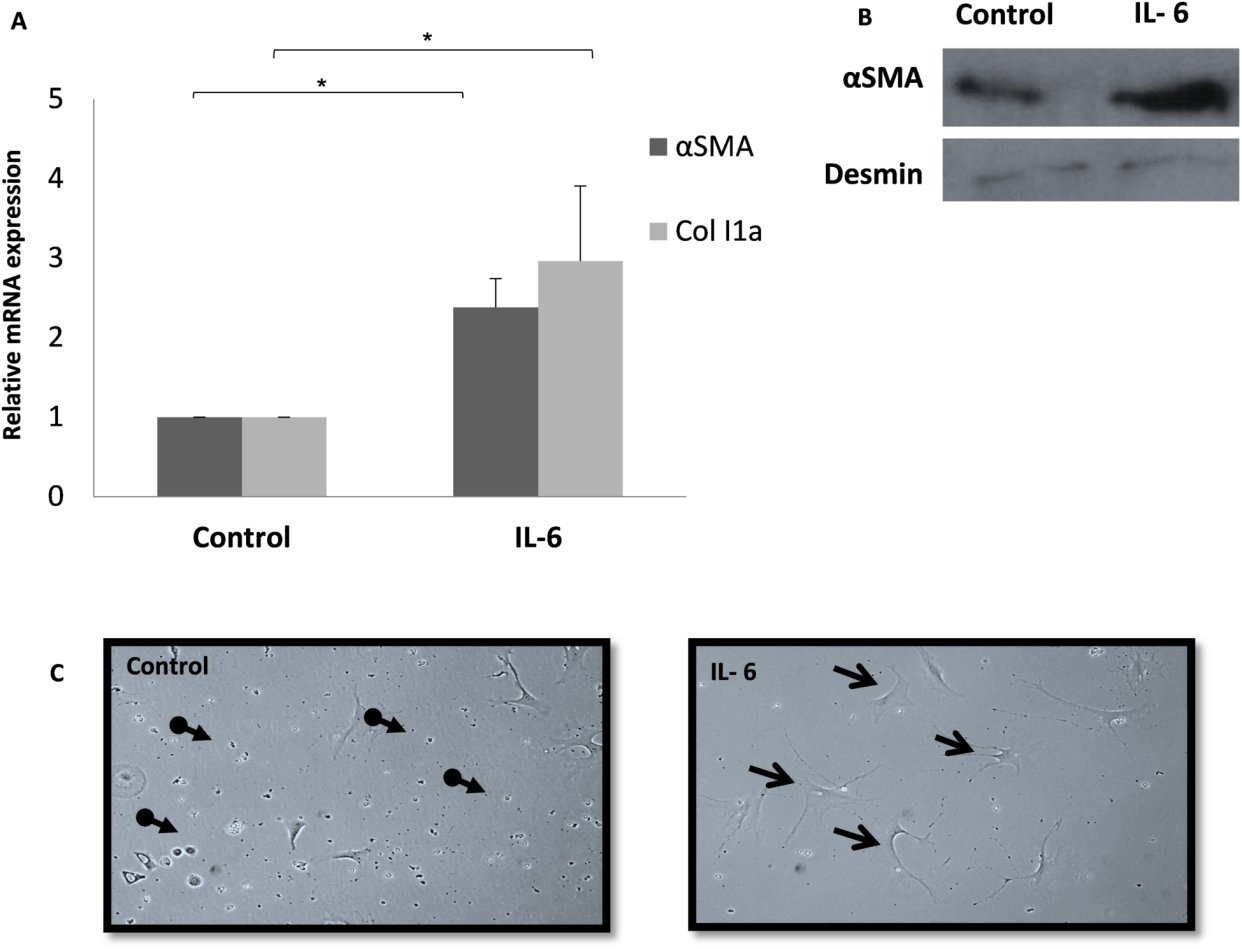
Alterations in HSCs characteristics following treatment with IL6. Freshly isolated primary HSCs were treated with 100 ng/ml IL-6 in 2% FBS DMEM. HSCs were harvested 24 h later and the levels of αSMA and Col1a were evaluated using qRT-PCR. qPCR relative mRNA expression levels are shown. Error bars indicate standard deviation, * *p* < 0.05. (A). Protein lysates prepared after 96 h exposure to 100 ng/ml IL-6, were subjected to Western blotting using antibodies for αSMA, and desmin as a reference gene (B). Representative light microscopy images of HSCs treated with 100 ng/ml IL-6 for 96 h (C) Non-activated HSC cells are marked with the broken line arrow and activated cells are marked with the full line arrow.

Freshly isolated primary HSCs treated for 24 h with increasing concentrations of purified IL-6 demonstrated markers of activation, as manifested by the up-regulation of αSMA and Col1a expressions levels (Fig 1). To further characterize the effect of IL-6 on the activation of HSCs, cells were treated with 100 ng/ml IL-6, for 24 h and 96 h. αSMA and Col1a mRNA levels were significantly upregulated after treatment with IL-6 for 24 hours (Fig 2A) and αSMA protein levels were higher in HSCs treated with 100 ng/ml IL-6 for 96 hours as compared to the Desmin that serves as a reference gene (Fig 2B). In parallel, drastic morphological changes were noted, with the appearance of a spindle-like fibroblast, resembling a profibrogenic phenotype, as compared to the untreated HSCs, which remained quiescent (Fig 2C).

### Both JAK/STAT and MAPK signaling pathway are necessary in the IL6 direct activation of HSCs

**Fig 3:**
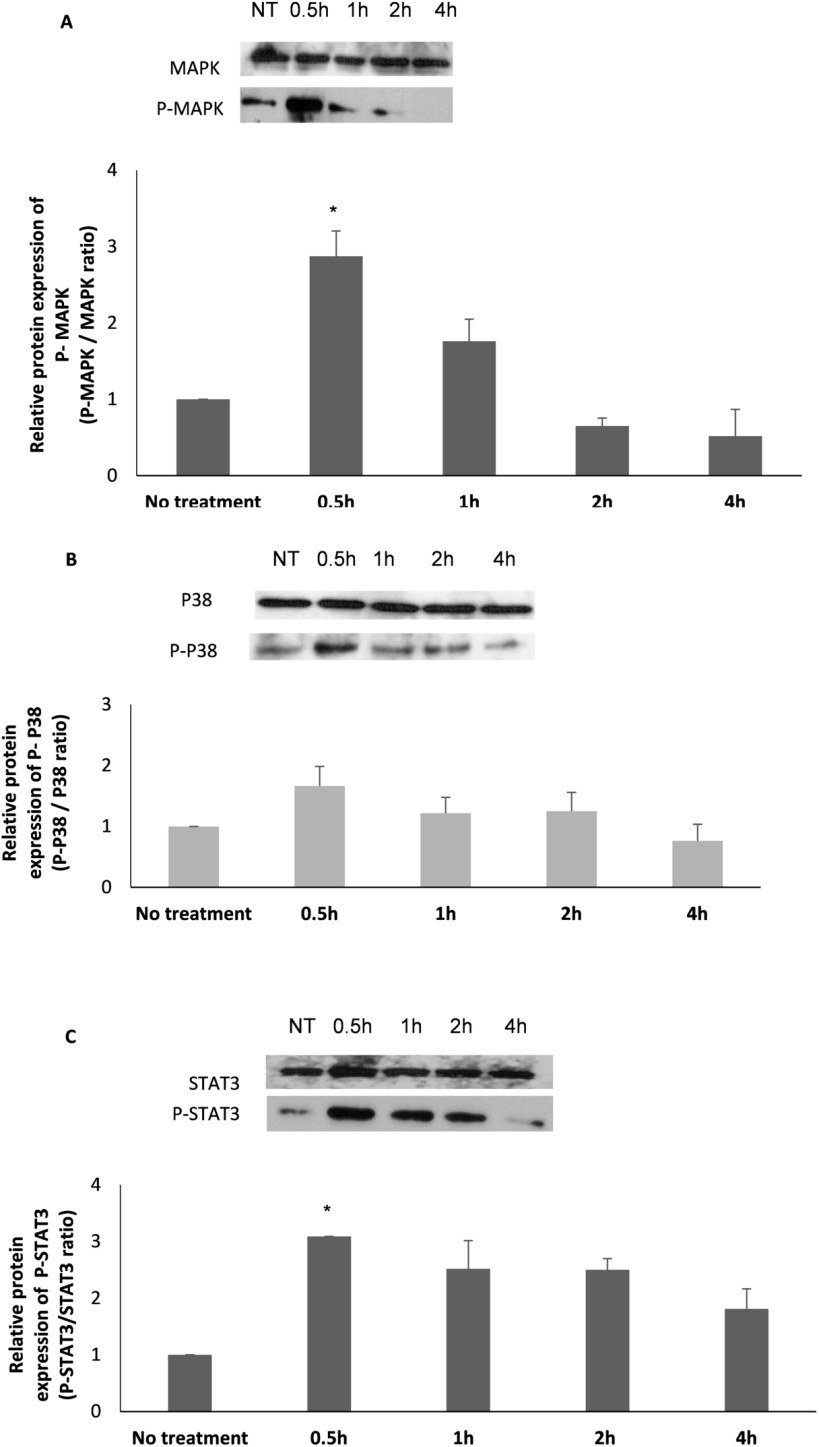
Both JAK/STAT and MAPK pathways are activated in IL-6-treated HSCs. Representative Western blot and quantitative analysis of p-MAPK and MAPK (A), p-p38 and p-38 (B) and STAT3 and p-STAT3 (C) protein expression following treatment with 100 ng/ml IL-6 for 0.5 h, 1 h, 2 h and 4 h, or after no treatment (NT). Error bars indicate standard deviation, * *p* < 0.05. The means of three experiments are presented.

To assess the involvement of the MAPK and STAT3/JAK signaling pathways in the IL-6-dependent activation of HSCs, primary cells were isolated and treated with 100ng/ml IL-6, and proteins purified 0.5, 1, 2 and 4 hours thereafter were analyzed. While MAPK, p38 and STAT3 expression levels remained constant following IL-6 treatment (Fig 3A, 3B and 3C), Phosphorylation levels of these proteins (p-p38, p-MAPK and p-STAT3) significantly increased 30 min after treatment, followed by a decline in the phosphorylation level compared to total protein (Fig 3A, 3B and 3C). More specifically, a (2.87)-fold increase in the relative phosphorylation levels of p-MAPK/MAPK, (1.66)-fold increase in the relative phosphorylation levels of p-p38/p38, and (3.1)-fold increase in the relative phosphorylation levels of p-STAT3/STAT3 were noted 30 minutes after IL-6 administration, compared to untreated samples (NT). (Fig 3A, 3B and 3C) Baseline phosphorylation levels of MAPK and p38 were observed again within 2–4 h of IL-6 addition to the medium. In parallel, STAT3 phosphorylation levels declined, but it did not return to baseline levels.

**Fig 4:**
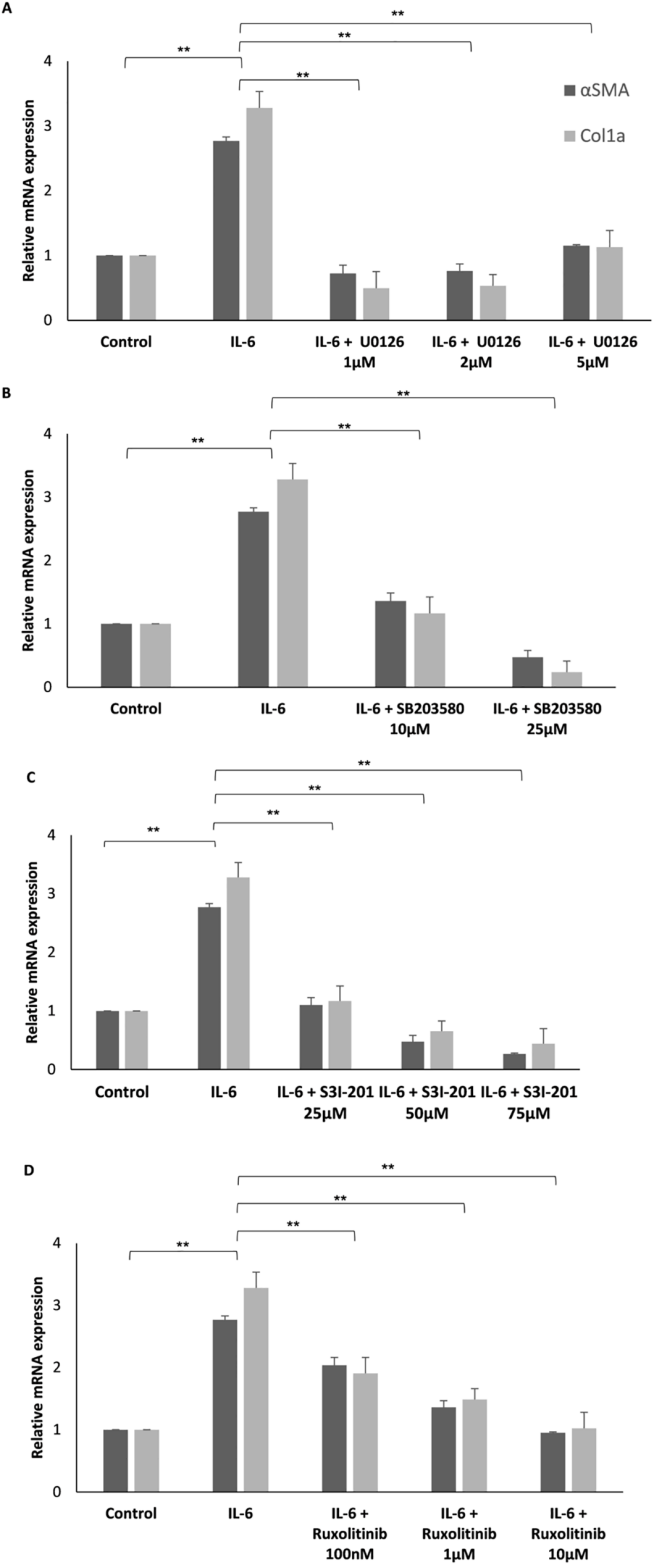
IL-6-dependent activation of HSCs is mediated by MAPK and JAK/STAT signaling pathways. Freshly isolated primary HSCs were treated with increasing concentrations of either U0126 (A) SB203580 (B), S3I-201 (C), or Ruxolitinib (D) for 1 hour, prior to the addition of 100 ng/ml IL-6 in 2% FBS DMEM. HSCs were harvested 24 hours later, RNA was purified and the levels of αSMA and Col1a were evaluated using qRT-PCR. qPCR relative mRNA expression levels are shown. Error bars indicate standard deviation, * *p* < 0.05, ** *p* < 0.01.

In order to determine the requisite for MAPK and JAK/STAT signaling pathways in the IL-6-mediated activation of HSCs, cells were treated with a specific signal transduction pathway inhibitor, 1 h prior to addition of 100ng/ml IL-6. Relative mRNA levels of Col1a and αSMA were measured in order to determine the activation levels of the HSCs. As expected 24 h after the addition of IL-6 only αSMA and Col1a levels indicated high levels of HSC activation. In contrast, relative mRNA levels in samples treated with IL-6 and either a specific MAPK, p38, JAK1/2 or STAT3 inhibitor were similar to control samples (Fig 4A, 4B, 4C and 4D). Each one of the inhibitors was added in increasing concentrations and the inhibition of HSC activation was concentration dependent and significant compared to the IL-6 activation alone (Fig 4A, 4B, 4C and 4D).

## Discussion

In our earlier report, we demonstrated that IL-6 mediates the direct activation of HSCs in hepatocyte-specific ADAR1 knock-out mice [15]. The current study further characterized this role and demonstrated that both the MAPK and JAK/STAT3 signal transduction pathways are required for IL-6 activity.

IL-6 is one of the most important pro-inflammatory cytokines, with pleiotropic functions in the body, including important roles in liver regeneration, as well as in metabolic function of the liver [7]. However, the role of IL-6 in liver fibrosis induction is still under debate [8,11–13], with most studies referring to pathological changes that occur in the liver after the in-vivo induction of hepatic fibrosis. Yet, little is known about the specific cell types that participate in this process, and the differential cell type-specific responses to IL-6 within the liver. Since HSCs play a crucial role in the development of liver fibrosis, we investigated the possibility of a direct effect of IL-6 on their activation and explored the signal transduction pathways involved in this process.

Using primary HSCs isolated from mouse livers, we showed that the administration of exogenous IL-6 upregulated the transcription levels of both αSMA and Col1a, increased αSMA protein levels and triggered phenotypical changes indicative of HSCs differentiation toward myofibroblast-like cells. IL-6 binds to the gp80/IL-6 receptor on hepatocytes, which then complexes with the signal transducer gp130. Binding of gp130 leads to dimerization of the intracellular domains of two gp130 molecules, which promotes association with receptor-associated JAKs. These enzymes then phosphorylate STAT molecules, which subsequently translocate to the nucleus and initiate gene transcription. Engagement of gp130 also leads to the recruitment of the cytoplasmic protein tyrosine phosphatase SHP2, which activates the Ras-ERK1/2 MAPK pathway [16]. The STAT3 signaling pathway is involved in many processes in the liver, such as induction of the acute phase response, protection against hepatocellular damage and promotion of liver regeneration [17]. However, its role in liver fibrogenesis remains largely unknown. The protein expression and phosphorylation levels of STAT3 were not altered in livers of HCV-associated fibrosis and alcoholic cirrhosis patients, however, STAT3-DNA binding was markedly suppressed when compared to healthy livers [18]. The hepatocyte-specific STAT3-depleted mouse model was associated with higher levels of liver injury, inflammation, fibrosis, and oxidative DNA damage compared with wild-type mice [19–22], indicating that hepatocyte STAT3 is important for liver protection against fibrosis induction. However, we present contradictory findings, where STAT3 signaling was necessary for the induction of IL-6-dependent HSC stimulation, while its blockage prevented HSC stimulation, which is suggested to lead to attenuation of the fibrotic process. Thus, we suggest that, STAT3 has a distinct and even contradictory role in different cell types.

In vivo studies have shown that stress-activated MAPK signaling converging at JNK and p38, plays a central role in inflammation-mediated liver injury and compensatory hepatocyte proliferation, leading to development of hepatocellular carcinoma (HCC) [23]. Furthermore, the MAPK signaling pathway was reported to be involved in the development of NAFLD [24]. A recent study demonstrated the involvement of TLR5-mediated MAPK signaling in CCL4-induced liver fibrosis development, as manifested by enhanced collagen accumulation and inflammatory infiltration in HSCs [25]. These studies indicate that, MAPK signaling serves as a stimulator of the inflammatory, fibrotic processes that may underlie hepatic destruction. Our results showing that IL-6 stimulation of the MAPK pathway, specifically through p38 and ERK, leads to the activation of HSC differentiation toward myo-fibroblast-like cells, agree with this notion.

The crosstalk between the STAT3/JAK and MAPK pathways is well established [26]. In 1998, it was shown that Jak1 is required for the tyrosine phosphorylation of SHP2 and, consequently, for the activation of the MAPK pathway [27]. Furthermore, it was demonstrated that p38MAPK is involved in IL-6-induced transcriptional activation of the STAT3 oncogene [28]. Our results can not indicate whether IL6-driven HSC activation involves each pathway independently, or whether there is crosstalk between the two signal transduction pathways. Future studies are warranted to pursue this aspect.

In conclusion, we have shown that IL-6 can directly induce the transition of HSCs toward myofibroblast-like cells displaying a fibrogenic phenotype. IL-6 activity is mediated via the MAPK and JAK/STAT signaling pathways, where elimination of either one inhibits the progression of the process. These findings might pave the road toward the development of potential therapeutic interventions for hepatic fibrosis.
